# mTORC1 accelerates osteosarcoma progression via m^6^A-dependent stabilization of USP7 mRNA

**DOI:** 10.1038/s41420-024-01893-9

**Published:** 2024-03-11

**Authors:** Zhengming Yang, Wei Yu, Ankai Xu, Bing Liu, Libin Jin, Huimin Tao, Dimin Wang

**Affiliations:** 1 Department of Orthopedics, 2nd Affiliated Hospital, School of Medicine, Zhejiang University, Hangzhou, China; 2 Orthopedics Research Institute of Zhejiang University, Hangzhou, China; 3 Department of Reproductive endocrinology, School of Medicine, Zhejiang University, Hangzhou, China

**Keywords:** Bone cancer, Epigenetics

## Abstract

Osteosarcoma (OS) is considered a sex steroid hormone-dependent bone tumor. The development and progression of OS are regulated by 17β-estradiol (E2). However, the detailed mechanisms of E2-modulated OS progression remained to be elucidated. Here, we found that E2-activated mammalian target of rapamycin (mTOR) signaling promoted N6-methyladenosine (m^6^A) modification through regulating WTAP. Inhibition of mTOR complex 1 (mTORC1) reversed E2-activated WTAP expression. Meanwhile, inhibition of mTORC1 suppressed OS cell proliferation and migration. Deficiency of TSC2 activated mTORC1 signaling and enhanced OS cell proliferation and migration, while abrogated by Rapamycin. Interestingly, mTOMC1 promoted mRNA stability of ubiquitin-specific protease 7 (USP7) through m^6^A modification. Loss of USP7 suppressed the proliferation, migration, and ASC specks, while promoted apoptosis of OS cells. USP7 interacted with NLRP3 and deubiquitinated NLRP3 through K48-ubiquitination. USP7 was upregulated and positive correlation with NLRP3 in OS patients with high level of E2. Loss of USP7 suppressed the progression of OS via inhibiting NLRP3 inflammasome signaling pathway. Our results demonstrated that E2-activtated mTORC1 promoted USP7 stability, which promoted OS cell proliferation and migration via upregulating NLRP3 expression and enhancing NLRP3 inflammasome signaling pathway. These results discover a novel mechanism of E2 regulating OS progression and provide a promising therapeutic target for OS progression.

## Introduction

Osteosarcoma (OS) is a skeletal sarcoma occurring in the metaphysis or craniofacial bones. During the last few years, the morbidity and mortality of OS have been rising [1]. The incidence of metastasis is 80% in the initial diagnosis of osteosarcoma [2]. The 3-year survival rate with distant metastasis of OS is less than 30% [3]. The latest study stated that the treatment methods for OS have been developed from surgery to neoadjuvant chemotherapy, radiotherapy, limb salvage treatment, molecular targeted therapy, immunotherapy, ablation treatment, and stem cell therapy [4]. There has been significant improve in the treatment of OS, but the treatment efficacy remains to be improved owing to the rapid deterioration and metastasis in OS.

17β-estradiol (E2) is one of the main estrogens, which not only plays important roles in reproductive organs, but also contains other tissues like bone, liver, and brain, dependently or independently through estrogen receptors α or β [5, 6]. A previous study reported that E2 protected human U2OS cells from death by expressing estrogen receptors α and β [7]. A new research makes known that E2 supplementation induced OS in mice [8]. Mammalian target of rapamycin complex 1 (mTORC1) is composed of mTOR, Raptor, mLST8 and Deptor [9]. Extensive previous research has shown that mTOR is aberrantly activated in OS, which accelerate the growth and metastasis of OS, and suppressed the apoptosis in OS [10, 11]. A latest study showed that mTORC1 was activated in OS clinically [12]. A previous study displayed that phosphorylated ribosomal S6 kinase 1 (p-S6K1) protein upregulation led to mTORC1 activation by E2 in chondrocytes [13]. Nevertheless, the link between mTORC1 and E2, as well as the mechanism involves in OS progression remains unknown.

N6-methyladenosine (m^6^A), methylation modification of adenosine N6 sites, mainly affects mRNA variable splicing, transport, translation, degradation, and stabolity [14]. m^6^A modification plays an important regulatory function, which involves in a variety of cell physiology process and multiple diseases, including obesity, diabetes, and various malignancies [15–17]. Previous studies have shown that m^6^A modification were dysregulated in the progression of osteosarcoma, which involved in the development of osteosarcoma [18, 19]. Recent studies found that mTORC1 regulated cell growth through enhancement of m^6^A modification [20, 21].

Ubiquitin-specific protease 7 (USP7) is a deubiquitination enzyme that prevents the substrates from degradation [22, 23]. USP7 regulates proteins or genes involved in tumor suppression, DNA damage response, DNA replication, viral infections, and epigenetics [24, 25]. Previous studies showed that the dysfunction of USP7 related to multiple human malignancies such as breast, lung, and cervical cancers [24, 26]. A recent study stated that USP7 was upregulated in OS tissues, and involved in OS cell invasion and migration [27]. NLRP3 inflammasome contains NLRP3, ASC, and caspase-1, which induces cell death and inflammation by responding to infection and stress [28, 29]. Recent studies have demonstrated that NLRP3 inflammasome activation was regulated by USP7 through NOX4/ROS pathway or SOX9/miR-96-5p pathway in NLRP3 inflammasome-associated inflammatory disorders [30, 31]. The suppression of USP7 in macrophages demonstrated a blockage of NLRP3 inflammasome activation [32]. However, the role of USP7 and NLRP3 inflammasome in OS remains to be explored. In this study, we found that E2 activated mTORC1 signaling and promoted the stability of USP7 through enhancement of m^6^A levels of CDS region. USP7 deubiquitinated NLRP3 through K48-ubiquitination to increase the NLRP3 expression and the ASC speck formation. Our results demonstrated that E2-activtated mTORC1 promoted USP7 stability, which promoted OS cell proliferation and migration via upregulating NLRP3 expression and NLRP3 inflammasome signaling pathway. These results discover a novel mechanism of E2 regulating OS progression and provide a promising therapeutic target for OS progression.

## Results

### E2 activated mTOR-mediated m^6^A modification

**Fig. 1:**
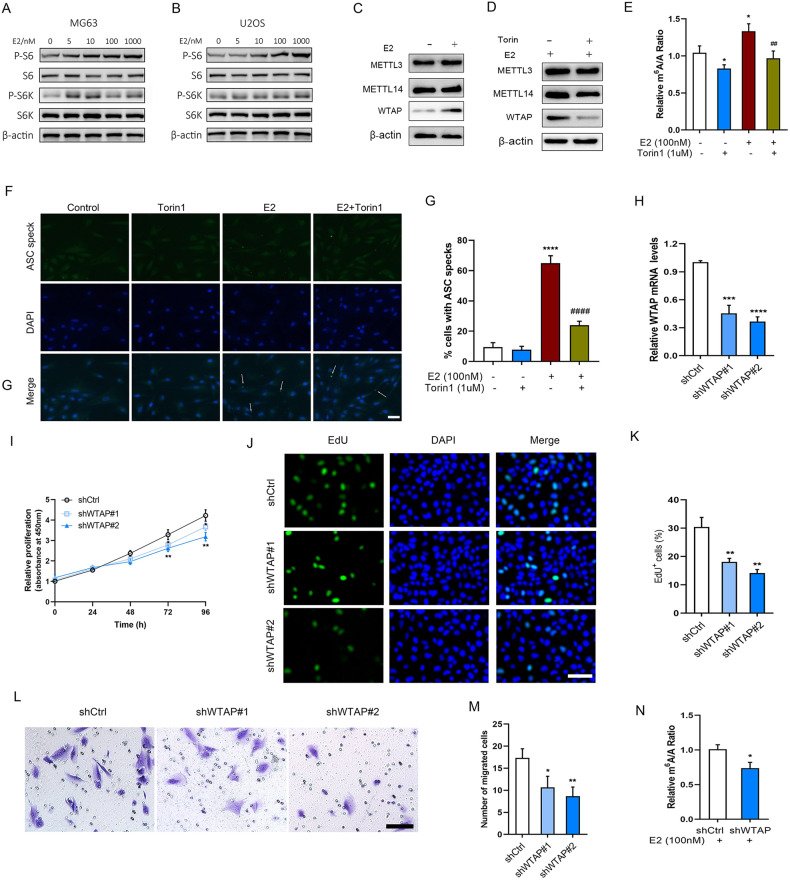
E2 activated mTOR-mediated m^6^A modification. **A**, **B** The protein expression levels of p-S6, S6, p-S6K, and S6K in MG63 and U2OS cells after treatment with 0–1000 nM of E2 for 48 h. **C** The protein expression levels of MTTLE3, METTL14, and WTAP in U2OS cells after treatment with or without 100 nM of E2 for 48 h. **D** The protein expression levels of MTTLE3, METTL14, and WTAP in U2OS cells after treatment with 100 nM of E2 for 48 h with or without pretreatment of 1 μM of torin1. **E** LC–MS analysis of m6A levels in U2OS cells treated with with 100 nM of E2 for 48 h with or without pretreatment of 1 μM of torin1. **F**, **G** Determination and quantification of ASC speck formation by immunofluorescence staining in 100 nM of E2 treated U2OS cells with or without torin1, scale bar: 50 µm. Arrows signify ASC speck. **H** The mRNA expression levels were determined following following shCtrl, shWTAP#1, and shWTAP#2 transfection. **I** The relative cell proliferation in shCtrl, shWTAP#1, and shWTAP#2 transfected U2OS cells for 0–96 h. The EdU staining (**J**) and EdU positive cells (**K**) in shCtrl, shWTAP#1, and shWTAP#2 transfected U2OS cells for 48 h, scale bar: 50 µm. **L**, **M** The migration capacity and migrated cell number in shCtrl, shWTAP#1, and shWTAP#2 transfected U2OS cells for 24 h, scale bar: 50 µm. **N** LC–MS analysis of m6A levels in U2OS cells treated with with 100 nM of E2 following shCtrl or shWTAP#2 transfection. *P < 0.05, **P < 0.01, ***P < 0.001, ****P < 0.0001 vs control group, ^##^*P* < 0.01, ^####^*P* < 0.0001 vs E2 group.

S6K is the downstream signal of mTOR, phosphorylates the downstream ribosomal protein S6 [33]. We found that the protein expression of p-S6 and p-S6K was obviously enhanced with increased concentrations of E2 treatment in U2OS and MG63 cells (Fig. 1A, B). Previous studies have shown that the mTOR signaling regulates m^6^A modification [20, 21]. To evaluate whether E2 and mTOR affect m^6^A modification, m^6^A levels and related proteins were determined following treatment with E2 with or without torin1 pretreatment. We found that E2 increased WTAP expression, but not METTL3 and METTL14, whereas Torin1 suppressed E2 increased WTAP expression (Fig. 1C, D). m^6^A levels were increased in E2 treated cells, while abrogated by Torin1 (Fig. 1E), suggested WTAP expression was associated with mTOR signaling regulating m^6^A modification. NLRP3 was revealed to be involved in OS progression [34]. We found the ASC speck was significantly increased in E2 treated cells compared with control, while abrogated by Torin1 (Fig. 1F, G). To further determine the effect of m^6^A modification on OS cell function, the proliferation and migration were evaluated in WTAP-knocked down cells. The downregulated effect of USP7 was testified by transfection with shWTAP#1 and shWTAP#2 (Fig. 1H). We found loss of WTAP significantly decreased proliferation of U2OS cells for 72 h treatment compared with control (Fig. 1I). The EdU positive cells were significantly decreased in WTAP-knocked down U2OS cells compared with control (Fig. 1J, K). Moreover, loss of WTAP significantly decreased migration cell number compared with control in U2OS cells (Fig. 1L, M). In addition, we found that m^6^A levels were decreased in WTAP-knocked down cells in the presence of E2 (Fig. 1N).

### mTORC1 regulated OS cell proliferation and migration

**Fig. 2:**
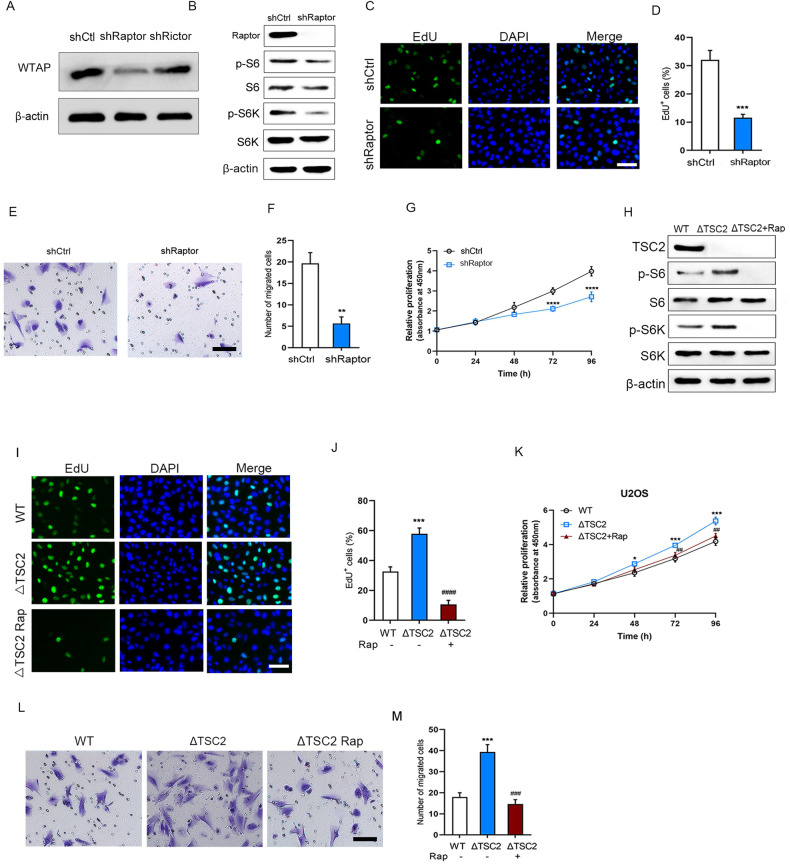
mTORC1 enhanced U2OS cell proliferation and migration. **A** Immunoblot analysis of WTAP in shCtrl, shRaptor, or shRictor transfected U2OS cells for 48 h. **B** Immunoblot analysis of Raptor, p-S6, p-S6K, in shCtrl, shRaptor, or shRictor transfected U2OS cells for 48 h. The EdU staining (**C**) and EdU positive cells (**D**) in shCtrl or shRaptor transfected U2OS cells for 48 h, scale bar: 50 µm. **E**, **F** The migration capacity and migrated cell number in shCtrl or shRaptor transfected U2OS cells for 24 h, scale bar: 50 µm. **G** The relative cell proliferation in shCtrl or shRaptor transfected U2OS cells for 0-96 h. **H** Immunoblot analysis of TSC2, p-S6, p-S6K, in WT or ΔTSC2 U2OS cells treated with or without rapamycin (100 nM). The EdU staining (**I**) and EdU positive cells (**J**) in WT or ΔTSC2 U2OS cells treated with or without rapamycin (100 nM), scale bar: 50 µm. **K** The relative cell proliferation in WT or ΔTSC2 U2OS cells treated with or without rapamycin (100 nM) for 0–96 h. **L**, **M** The migration capacity and migrated cell number in WT or ΔTSC2 U2OS cells treated with or without rapamycin (100 nM), scale bar: 50 µm. *P < 0.05, **P < 0.01, ***P < 0.001, ****P < 0.0001 vs control group, ^##^*P* < 0.01, ^###^*P* < 0.001, ^####^*P* < 0.0001 vs ΔTSC2 group.

To explore which mTOR complex modulates OS cell proliferation and migration, Raptor and Rictor were knocked down. We found loss of Raptor, but not Rictor reduced WTAP levels (Fig. 2A), suggesting that mTORC1 activates WTAP expression. The protein expression levels of p-S6 and p-S6K were decreased in Raptor-knocked down cells (Fig. 2B). Moreover, we found loss of Raptor significantly reduced EdU positive cells compared with control (Fig. 2C, D). The migrated cell number was significantly decreased in Raptor-knocked down cells compared with control (Fig. 2E, F). Loss of Raptor significantly decreased proliferation of U2OS cells for 72 h treatment compared with control (Fig. 2G). To further determine whether mTORC1 regulated OS cell proliferation and migration, the negative regulator of mTORC1 signaling TSC2 was knocked out. The protein expression levels of p-S6 and p-S6K were obviously upregulated in TSC2-knocked out cells, while abrogated by rapamycin, an inhibitor of mTORC1 (Fig. 2H). We found deficiency of TSC2 significantly increased EdU positive cells compared with control, while abrogated by rapamycin (Fig. 2I, J). Deficiency of TSC2 significantly increased proliferation of U2OS cells for 48 h treatment compared with control, while abrogated by rapamycin at 72 h (Fig. 2K). The migration capacity was significantly enhanced in TSC2-knocked out cells compared with control, while abrogated by rapamycin (Fig. 2L, M).

### mTOMC1 regulated mRNA stability of USP7 through m^6^A modification

**Fig. 3:**
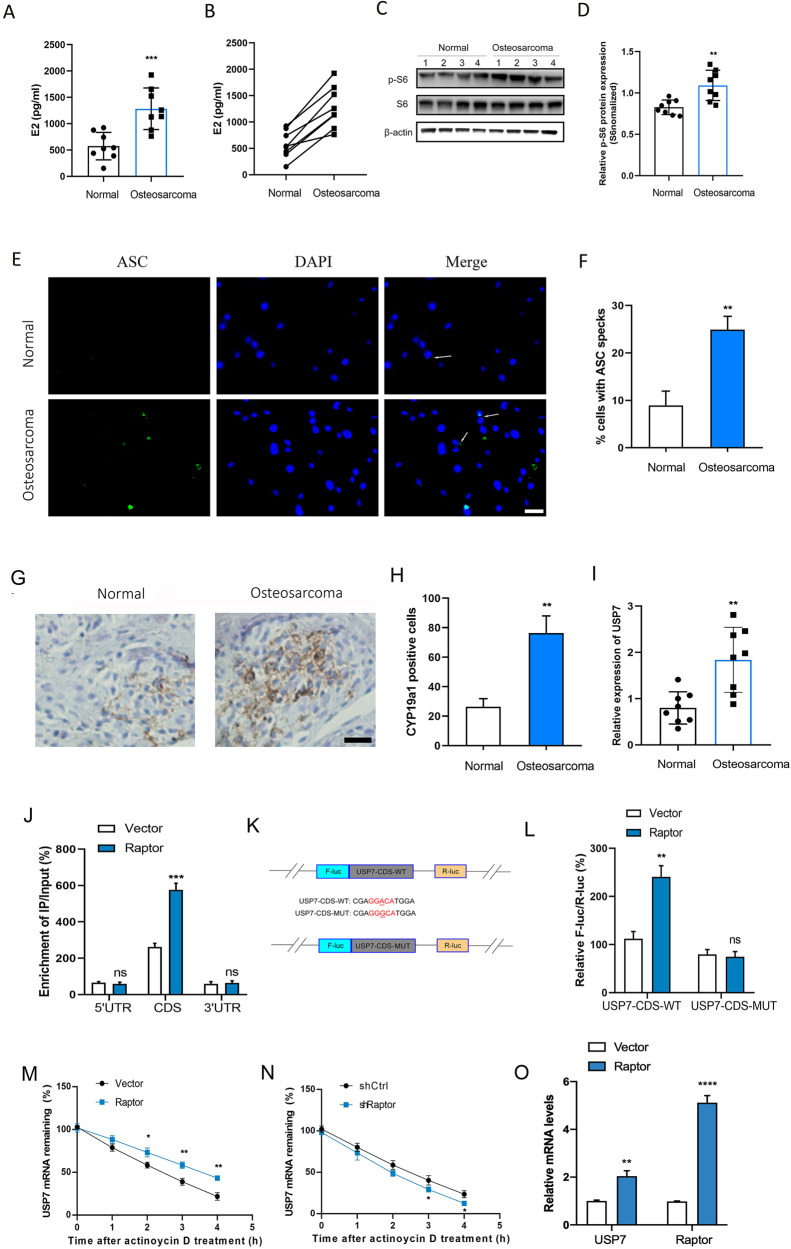
mTOMC1 regulated mRNA stability of USP7 through m^6^A modification. **A** Eight patients with high levels of E2 were collected. The E2 content in 8 OS tissues or normal tissues. **B** The corresponding E2 content between each OS tissues and normal tissues. **C** Immunoblot analysis of p-S6, and S6 in 8 OS tissues or normal tissues. **D** Quauntification of p-S6 protein expression in OS tissues or normal tissues (*n* = 8). **E**, **F** The formation and quantification of ASC speck in OS tissues or normal tissues (*n* = 8). **G** IHC analysis of the expression of CYP19a1 in OS tissues or normal tissues, scale bar: 50 µm. **H** Quantification analysis of CYP19a1 positive cells in OS tissues or normal tissues. **I** The relative expression of USP7 in OS tissues and normal tissues (*n* = 8). **J** The m^6^AqPCR assay was performed to analyze the m^6^A enrichment in 5′UTR, CDS, and 3’UTR of USP7 mRNA in vector or Raptor transfected U2OS cells. **K** Schematic illustrations of mutation in m6A motif in CDS of USP7. **L** F-Luc/R-Luc activity in vector or Raptor transfected U2OS cells following treatment with USP7-CDS-WT or USP7-CDS-MUT. **M** Lifetime USP7 mRNA levels in vector or Raptor transfected U2OS cells following treatment with 0.5 mg/mL of actinomycin D for 0–4 h. **N** Lifetime USP7 mRNA levels in shCtrl or shRaptor transfected U2OS cells following treatment with 0.5 mg/mL of actinomycin D for 0–4 h. **O** The USP7 and Raptor mRNA levels in in vector or Raptor transfected U2OS cells. *P < 0.05, **P < 0.01, ***P < 0.001, ****P < 0.0001 vs control group. ns not significant.

To investigate the effect of mTOMC1 on OS progression, eight OS patients with high level of E2 were collected. The E2 content was significantly increased in eight OS tissues compared with adjacent normal tissues (Fig. 3A, B). The protein expression levels of p-S6 were significantly enhanced in eight OS tissues compared with adjacent normal tissues (Fig. 3C, D). The ASC speck was significantly increased in OS tissues compared with normal tissues (Fig. 3E, F). We found the CYP19a1 positive cells were significantly increased in OS tissues compared with adjacent normal tissues (Fig. 3G, H). Interestingly, we found the expression levels of USP7 were significantly increased in OS tissues compared with adjacent normal tissues (Fig. 3I). Moreover, the m6A motif was predicted in the CDS and 3′UTR of USP7. RNA fragments were immunoprecipitated with m^6^A to identify m6A methylation modification in USP7 mRNA. As a results, we found the m6A enrichment of USP7 CDS, but not 5′UTR or 3′UTR, was significantly increased with Raptor overexpression (Fig. 3J). To further explore the effect of m^6^A methylation in CDS region of USP7, luciferase reporters containing the WT or MUT USP7 CDS were constructed (Fig. 3K). We found the luciferase activity of USP7-CDS-MUT was barely changed between Raptor-overexpressing cells and vector control cells compared with USP7-CDS-WT (Fig. 3L). The half-life of USP7 transcripts was significantly increased in Raptor-overexpressing cells compared with vector control cells (Fig. 3M). However, the half-life of USP7 transcripts was significantly decreased in Raptor-kocked down cells compared with control (Fig. 3N). In addition, the USP7 mRNA was increased in Raptor-overexpressing cells compared with vector control cells (Fig. 3O). These results suggested that mTOMC1 promoted mRNA stability of USP7 through CDS m6A modification.

### Loss of USP7 suppressed the proliferation and migration of OS cells

**Fig. 4:**
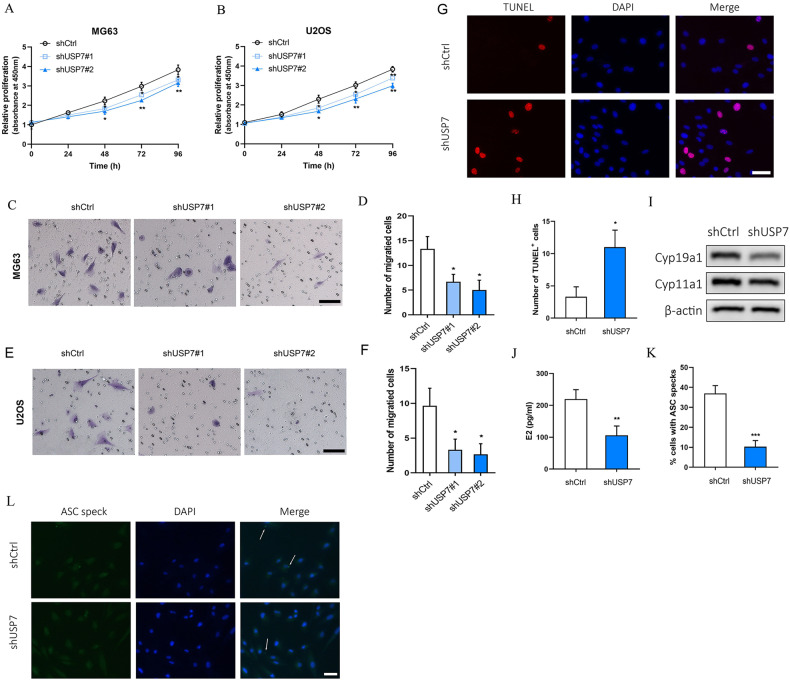
Loss of USP7 inhibited OS cell proliferation and migration. The relative cell proliferation in shCtrl, shUSP7#1 or shUSP7#2 transfected MG63 (**A**) and U2OS (**B**) cells for 0-96 h. **C**, **D** The migration capacity and migrated cell number in shCtrl, shUSP7#1 or shUSP7#2 transfected MG63 for 24 h, scale bar: 50 µm. **E**, **F** The migration capacity and migrated cell number in shCtrl, shUSP7#1 or shUSP7#2 transfected U2OS for 24 h, scale bar: 50 µm. **G**, **H** TUNEL staining and TUNEL positive cells in shCtrl or shUSP7#2 transfected U2OS for 24 h, scale bar: 50 µm. **I** The Cyp19a1 and Cyp11a1 protein expression levels in shCtrl or shUSP7#2 transfected U2OS cells for 48 h. **J** The E2 content in shCtrl or shUSP7#2 transfected U2OS cells for 48 h. **K**, **L** The formation and quantification of ASC speck in U2OS cells transfected with shCtrl or shUSP7#2, scale bar: 50 µm. Arrows imply ASC speck. *P < 0.05, **P < 0.01, ***P < 0.001 vs control group.

To further investigated the role of USP7 in the proliferation and migration of OS cells, USP7 was downregulated by transfection of shUSP7. The downregulated expression of USP7 was verified by transfection with shUSP7#1 and shUSP7#2 in Supplementary Fig. 1A. We found that the proliferation was significantly decreased in the USP7-knocked down cells compared with control (Fig. 4A, B). The migration capacity was significantly reduced in USP7-knocked down MG63 (Fig. 4C, D) cells and U2OS cells (Fig. 4E, F) compared with control. To further investigate the effect of USP7 on OS cells function, we analyzed the apoptosis and ASC specks in U2OS cells. The result showed that the TUNEL positive cells were markedly increased in USP7-knocked down cells compared with control (Fig. 4G, H). The aromatase CYP19a1 and CYP11a1 are key players in E2 synthesis pathway [35]. Interestingly, The expression levels of CYP19a1 and CYP11a1 were reduced in USP7-knocked down cells compared with control (Fig. 4I). Moreover, we found that E2 content was significantly decreased in USP7 downregulated cells compared with control (Fig. 4J). In addition, ASC specks were significantly reduced in USP7-knocked down cells compared with control (Fig. 4K, L).

### USP7 stabilized NLRP3 via decreasing K48-linked ubiquitination of NLRP3

**Fig. 5:**
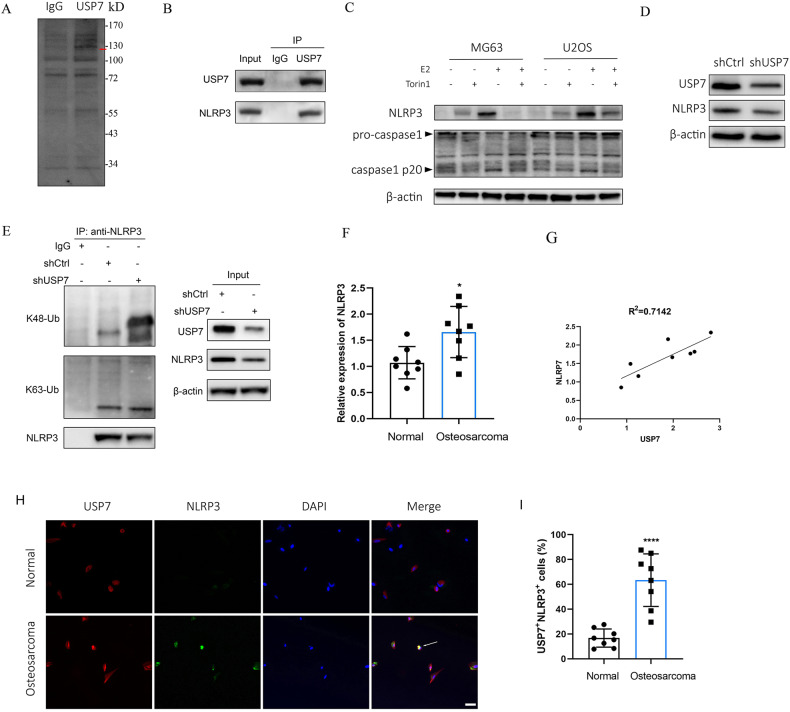
USP7 stabilizes NLRP3 via decreasing K48-linked ubiquitination of NLRP3. **A** The SDS-PAGE band pattern of immunoprecipitated proteins using anti-USP7 and IgG. The bands of protein were excised to mass spectrometry (MS) analysis for identification. The red arrow marks the distinct band (NLRP3) in USP7 immunoprecipitated samples. **B** The immunoprecipitated protein of NLRP3 were analyzed using anti-USP7 and IgG. **C** The protein expression levels of NLRP3, pro-caspase1, and caspase1 in MG63 and U2OS cells treated with 100 nM of E2 for 48 h with or without pretreatment of 1 μM of torin1. **D** The protein expression levels of NLRP3 and USP7 in U2OS cells treated with shCtrl or shUSP7 for 24 h. **E** U2OS cells were transfected with shCtrl or shUSP7 for 24 h. The protein extracts were immunoprecipitated with IgG beads of anti-NLRP3, and the protein expression levels of K48-ubiquitin, K63-ubiquitin and NLRP3 were detected. **F** The relative expression of NLRP3 in OS tissues and normal tissues (*n* = 8). **G** The correlation between USP7 and NLRP3 in OS tissues or normal tissues (*n* = 8). **H** The colabel staining of USP7 and NLRP3 in OS tissues or normal tissues, scale bar: 50 µm. Arrow indicates USP7^+^NLRP3^+^ cells. **I** Quantification of the USP7^+^NLRP3^+^ cells in OS tissues or normal tissues (*n* = 8). **P < 0.01, ****P < 0.0001.

To explore the mechanism of USP7 regulating OS function, Mass spectrometry (MS) and co-immunoprecipitation (Co-IP) assay were used to determine potential interaction targets of USP7 in OS cells. The Co-IP samples were resolved in an SDS-PAGE gel, demonstrating the distinct pattern of USP7-interacting proteins, as well as overlapping bands in the Co-IP samples (Fig. 5A). The distinct proteins were identified by MS and the results revealed that NLRP3 was a interaction partner for USP7. We then validated the interactions of USP7 with NLRP3 by Co-IP and western blot. The results showed that USP7 interacted with NLRP3 (Fig. 5B). Additionally, 56 proteins were identified in the USP7 immunoprecipitates by IP-MS (Supplementary Table 1). Moreover, the levels of NLRP3 and caspase1 were measured in E2 treated cells with or without Torin1. The NLRP3 expression levels were apparently increased in E2 treated MG63 and U2OS cells, while abrogated by Torin1 (Fig. 5C). The expression of pro-caspase 1 was barely changed in E2 treated cells compared with control, whereas caspase 1 p20 was increased in E2 treated cells compared with control, which abrogated by Torin1 (Fig. 5C). The protein expression of NLRP3 was obviously decreased in USP7-knocked down cells compared with control (Fig. 5D). Moreover, we explored whether USP7 interacted NLRP3 by deubiquitination. We evaluated the K48-and K63-ubiquitin levels of NLRP3 following downregulation of USP7. The results revealed that K48-ubiquitination of NLRP3 increased after downregulation of USP7, whereas K63-ubiquitination barely changed (Fig. 5E). These results suggest that downregulation of USP7 increased K48-ubiquitination of NLRP3, thereby decreasing the expression of NLRP3. Consistently, the expression levels of NLRP3 were increased in OS tissues compared with normal tissues (Fig. 5F). We found the positive correlation between USP7 and NLRP3 in OS tissues (Fig. 5G). Meanwhile, we found that USP7 staining was colabled with NLRP3 staining and the proportion of USP7^+^NLRP3^+^ cells were significantly increased in OS tissues compared with control tissues (Fig. 5H, I), which suggested that USP7 interacted with NLRP3 in OS. In addition, we also found the fluorescence intensity of USP7 was significantly enhanced in OS tissues compared with control tissues (Fig. 5H, Supplementary Fig. 1B).

### Loss of USP7 or Raptor suppressed OS tumor growth in vivo

**Fig. 6:**
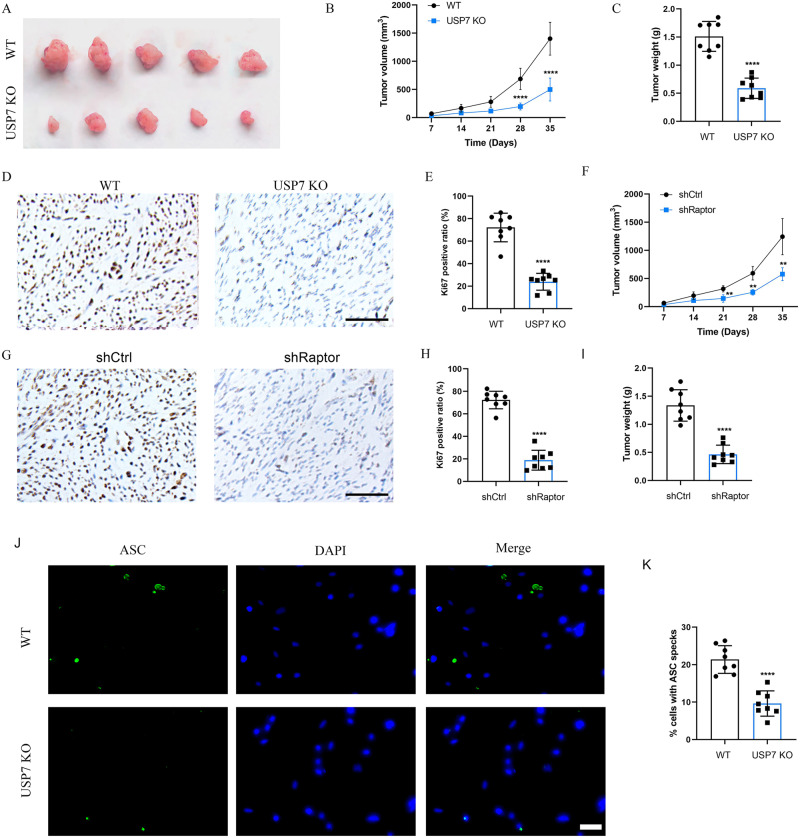
Knockout of USP7 or downregulation of mTORC1 suppressed tumor growth of OS cells in vivo. **A** The flank of the nude mice were subcutaneously injected with 1 × 10^6^ of USP7 KO and control cells. Tumor volume (**B**) and tumor weight (**C**) were calculated and observed (*n* = 5). **D**, **E** IHC analysis and quantification of the expression of Ki-67 in USP7 KO or control mice (*n* = 5), scale bar: 100 µm. **F** The flank of the nude mice were subcutaneously injected with 1 × 10^6^ of shCtrl or shRaptor treated U2OS cells. Tumor volume was calculated (*n* = 5). **G**, **H** IHC analysis and quantification of the expression of Ki-67 in shCtrl or shRaptor treated mice (*n* = 5), scale bar: 100 µm. **I** Tumor weight was collected in shCtrl or shRaptor treated mice (*n* = 5). **J**, **K** The formation and quantification of ASC speck in shCtrl or shRaptor treated mice, scale bar: 50 µm. **P < 0.01, ****P < 0.0001 vs control group.

**Fig. 7:**
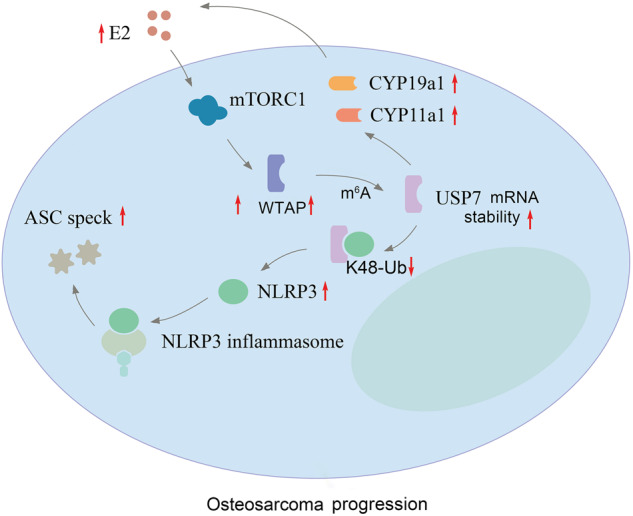
The mechanism of mTORC1 regulated USP7/NLRP3 signaling in OS progression. E2-activated mTORC1 promotes stabilization of USP7 through m^6^A modification. USP7 interacts directly with NLRP3 and deubiquitinases NLRP3. The ASC speck is increased following the assembling and activating of NLRP3 inflammasome, which leads to activation of caspase1.

To evaluate the effect of USP7 on OS growth in vivo, we examined knockout of USP7 (USP7 KO) using a tumor-bearing model. The flank of the nude mice was subcutaneously injected with 1 × 10^6^ of USP7 KO and control U2OS cells. Comparing USP7 KO mice to the control mice, we found a significant reduction in tumor growth (Fig. 6A). In contrast to the control group, USP7 KO effectively reduced the volumes and weights of tumors implanted in nude mice (Fig. 6B, C). Importantly, the Ki67 positive ratios were significantly decreased in USP7 KO mice compared with control mice (Fig. 6D, E). Moreover, we found the volumes and weights of tumors were significantly reduced in Raptor-knocked down mice compared with control mice (Supplementary Fig. 1C, Fig. 6F, I). The Ki67 positive ratios were significantly decreased in Raptor-knocked down mice compared with control mice (Fig. 6G, H). To investigate the effect of USP7 on ASC speck in vivo, ASC specks were stained in USP7 KO mice tissues. The results showed that the proportion of ASC specks was significantly decreased in USP7 KO mice compared with control mice (Fig. 6J, K). These results were consistent with the findings in OS cells that mTORC1-stabilized USP7 activated NLRP3 inflammasome pathway (Fig. 7), which was involved in OS progression.

## Discussion

OS is a malignant bone tumors which is regulated by sex steroid hormones and mostly affects children and adolescents during the period of large hormones secretion [36, 37]. A previous study showed that E2 promoted the migration, invasion, and proliferation in U2OS cells through upregulation metastasis-associated lung adenocarcinoma transcript 1 (MALAT1) expression by Sp1 [38]. Another study showed that E2 led to a poor prognosis of lung cancer by downregulation of p53 and increased M2 macrophages polarization [39]. In this study, we found that the protein expression of p-S6 and p-S6K was increased with E2 treatment in U2OS and MG63 cells. Previous studies have shown that the mTOR signaling regulates m^6^A modification [20, 21]. We found that E2 increased WTAP expression, but not METTL3 and METTL14, whereas Torin1 suppressed E2 increased WTAP expression, suggested that mTOR regulates m^6^A modification through upregulating WTAP. Moreover, we found that inhibition of mTORC1 suppressed the proliferation and migration of OS cells, which controlled by E2. Furthermore, we found that the ASC speck was significantly increased in the treatment of E2, whereas Torin1 abrogated the effect of E2 on ASC speck, which suggested that the E2 activated NLRP3 inflammasome through the mTOR signaling in OS cells.

The activated mTORC1 phosphorylated two downstream effectors, S6K1 and 4E-BP1 [40]. It has been proved that the growth and proliferation of OS cells are inhibited by The downregulation of phosphorylated S6K1 and 4EBP1 [41]. The activated S6K1 directly phosphorylated the substrate at serine sites. According to a recent study, S6K directly phosphorylated phosphatase and tensin homolog deleted on chromosome ten (PTEN) at S380 following activation [42]. The activity of mTORC1 has been found to be regulated by growth factors, phosphatidic, insulin, leucine, and oxidative stress [43, 44]. Our study demonstrated that the activity of mTORC1 was induced by E2, and the stability of USP7 was enhanced by the activated mTORC1 upon E2 stimulation, which promoted USP7 deubiquitination function in OS cells. We also revealed that loss of USP7 inhibited the E2 content and enzymes Cyp19a1 and Cyp11a1, which suggested that mTORC1 stabilized USP7 may positive regulate E2 synthesis in osteosarcoma.

NLRP3 is a sensor protein that detects endogenous and/or external danger signals in the upstream of NLRP3 inflammasome, and the adaptor protein ASC is necessary for inflammasome assembly and activation [45]. It has been stated that deubiquitinases control the assembly and activation of NLRP3 inflammasome, including A20, USP50 and BRCC3 [46–48]. A previous study revealed that USP7 and USP47 activated NLRP3 inflammasome by regulating ASC oligomerization, NLRP3 ubiquitination, and speck formation in macrophages, but remained unclear whether USP7 and USP47 contributed to NLRP3 deubiquitination directly or indirectly [32]. We clarified that USP7 directly interacted with NLRP3 by K-48 deubiquitination. Moreover, the NLRP3 expression and ASC speck formation were reduced by downregulation of USP7, which improved the growth of OS cells as well. Thus, our study elucidated a novel mechanism of NLRP3 inflammasome activation that was regulated by deubiquitination of stabilized USP7 in OS cells.

In summary, our study demonstrated that the activation of mTORC1 was induced by E2 in OS cells, which stabilized the mRNA of USP7 by inducing m6A modification on USP7 CDS region. Inhibition of mTORC1 suppressed OS cell proliferation and migration. The NLRP3 was upregulated by USP7-mediated K48-deubiquitination, which stimulated NLRP3 inflammasome. Moreover, we found that loss of USP7 suppressed the progression of OS via inhibiting NLRP3 inflammasome signaling pathway. Our results demonstrated that E2-activtated mTORC1 promoted USP7 stability, which promoted OS cell proliferation and migration via upregulating NLRP3 expression and NLRP3 inflammasome signaling pathway. These results discover a novel mechanism of E2 regulating OS progression and provide a promising therapeutic target for OS progression.

## Methods

### Cell culture

U2OS and MG63 two human OS cells were bought from the Cell Bank, Chinese Academy of Sciences (Shanghai, China). The authentication of U2OS and MG63 cell lines was performed via comparisons with the STR database. The cultivation conditions for U2OS and MG63 are DMEM medium (Gibco) with 10% fetal bovine serum (Gibco) and the incubation conditions contain 37°C with 5% CO_2_ and 100% humidity.

### Clinical specimen

Pathological analysis of OS tissues and matched adjacent normal tissues was conducted on clinical samples taken from OS patients. A total of 8 OS patients with high level of E2 were enrolled in the study. The histological diagnosis was performed blindly by two experienced pathologists. Inclusion and exclusion criteria: surgically removed OS tissues and adjacent noncancerous tissues were collected from these patients before the commencement of chemotherapy or radiotherapy. All participants have signed written informed consent at the Second Affiliated Hospital of Zhejiang University (Hangzhou, China), which was approved by the Medical Ethics Committee of the Second Affiliated Hospital of Zhejiang University (No. 20220956).

### Plasmids

The USP7, WTAP, and Raptor shRNAs were cloned into the pLKO lentiviral vector (You Bio, China). Control vectors carried scrambled non-targeting shRNAs (shCtrl). Raptor sequence was cloned in pLVX lentiviral vector (You Bio, China). Plenty vectors were used as control. The calcium phosphate technique is used to transfect the HEK-293T packaging cells. Lentiviral particles from single plasmids were produced and viral titer was calculated. Puromycin (2 mg/mL) was added 48 h post infection and surviving cells were harvested after 3 days.

### Western blotting

The MG63 and U2OS cells were lysed after washing with cold PBS to extract the total proteins. Protein quantification was done by using the BCA Protein Assay Kit (Beyotime, China). The proteins were separated by 10% sodium dodecyl sulfate-polyacrylamide gel electrophoresis (SDS-PAGE), next they were placed in nitrocellulose membrane (NC). The NC membranes were blocked at room temperature with 5% bovine serum albumin (BSA) for 1 h. The primary antibody, which includes p-S6 (1:1000, Cell Signaling Technology, Danvers, MA, USA), S6 (1:1000, Cell Signaling Technology), p-S6K (1:1000, Cell Signaling Technology), S6K (1:1000, Cell Signaling Technology), NLRP3 (1:1000, Abcam, Cambridge, UK), caspase1 (1:1000, Abcam), Raptor (1:1000, Cell Signaling Technology), TSC2 (1:1000, Cell Signaling Technology), WTAP (1:1000, Cell Signaling Technology), TSC2 (1:1000, Cell Signaling Technology), METTL3 (1:1000, Abcam), METTL14, (1:1000, Abcam), caspase1 (1:1000, Cell Signaling Technology), anti-K48 Ub (1:1000, Abcam), and anti-K63 Ub (1:1000, Abcam), cyp19a1 (1:1000, Proteintech, USA), cyp11a1 (1:1000, Abcam), USP7 (1:1000, Abcam), β-actin (1:1000, Cell Signaling Technology) were diluted by Tris-buffered saline (TBS), then they were incubated with the NC membranes after removing residual liquid at 4 °C overnight. Next, we incubated the secondary antibody (1:7500, Abcam) with the NC membranes at room temperature for 2 h. Finally, the chemiluminescence detection system was performed to visualize the protein bands, and used Image J software (NIH) to analyze.

### LC-MS analysis of m^6^A

Total RNA was isolated from OS tissues or cells by TRIzol (Invitrogen). The m^6^A levels were detected using quadrupole time-of-flight (QTOF) mass spectrometer (Agilent) in previous study [20]. Briefly, 100 ng mRNA was digested with 1 unit of nuclease P1 (Sigma) at 37 °C for 2 h, then incubated with 1 unit of alkaline phosphatase (Sigma) for 2 h at 37 °C. After incubation, the samples were run in solvent A and solvent B. MRM transitions were measured for adenosine and m^6^A. Concentrations of each compound in the samples were calculated using calibration curves constructed with standard compounds of adenosine (Abcam) and m^6^A (Abcam).

### Elisa

E2 was detected by a E2 ELISA kit (Elabscience). Briefly, tissues or cells were lysed to collect total proteins for ELISA assay. One hundred microliters diluted lysates were added to each well of the coated microplate, then incubated for 1 h at 37 °C. The different dilutions of standards were added for quantification. The plates were washed with 300 μL washing buffer 3 times, then 100 μL enzyme-linked polyclonal antibodies specific for E2 were added and incubated for 30 min at 37 °C in the dark, separately. The addition of 50 μL Substrate Reagent was used to reveal the reaction. After incubating until the color changed at 37 °C in dark, 100 μL stop solution was added. The OD value of each well was determined at a wavelength of 450 nm using a microplate reader (ELX800, BioTeK, USA).

### CCK-8 assay

The MG63 and U2OS cells were harvested to detect the ability of cell proliferation by CCK-8 (Dojindo, Kumamoto, Japan) based on the instructions provided by the manufacturer. Briefly, 100 μL cell suspension was seeded per well into 96-well culture plates. After incubation with shCtrl, shWTAP#1, shWTAP#2 or shRaptor for 0 h, 24 h, 48 h, 72 h, 96 h, added 10 μL CCK-8 solution and incubated at 37 °C for 4 h separately. Then, the absorbance at 450 nm was determined with a microplate reader (ELX800, BioTeK, USA).

### EdU staining assay

Cells were transfected with shCtrl, shWTAP#1, shWTAP#2 or shRaptor for 48 h. The cells were incubated with 50 µM of EdU (Ribobio) for 1 h. The cells were then fixed with 4% paraformaldehyde, and subjected to 0.5% Triton X-100 permeabilization. Then, the cells were stained with pollo®488 for 1 h. The cells were subsequently counterstained with DAPI and imaged via a fluorescence microscopy (Leica, Wetzlar, Germany).

### Transwell assay

The 100 μL diluted Matrigel (BD Biosciences, USA) was coated to the polycarbonate membrane on the upper chambers (BD Matrigel Invasion Chamber, BD Biosciences, USA). About 1 × 10^4^ OS cells were suspended in 200 μL medium without serum and seeded into the upper chambers of each transwell (8 μm pore size). We added medium compromising 10% FBS to the bottom chamber. After incubation with shCtrl, shWTAP#1, shWTAP#2, or shRaptor for 24 h, used cotton swabs to remove cells in the upper chamber, and 4% paraformaldehyde to fix the cells on the bottom chamber for 15 min, stained for 10 min using 0.1% crystal violet. An inverted light microscope was applied to count the migration cells (Olympus, Tokyo, Japan).

### Immunofluorescence and microscopy

The U2OS cells were seeded to the cover slides coated petri dishes. The cover slides were fixed by 4% paraformaldehyde after incubation with phosphate buffered saline (PBS), E2 (100 nmol/L), Torin1 (1 μM/L), E2+ Torin1 treatment for 24 h. 0.5% Triton X-100 was added for cell permeability for 20 min. After blocking, the slides or or OS tissue sections were incubated with the primary antibodies, anti-ASC speck (1:200, Santa Cruz, CA, USA) at 4 °C overnight, respectively. Then the slides were incubated with Alexa Fluor 488-conjugated secondary antibody (1:400, Life Techonologies, USA) for 30 min following washed by PBS for three times. Finally, the slides were counterstained with DAPI (Beyotime, China) and analyzed with a fluorescence microscope (Leica, Wetzlar, Germany). We used 4% paraformaldehyde to fix the OS tissues or normal tissues. After blocking, the slides were incubated with Alexa Fluor 594 conjugated USP7 antibody (1:200, Novus Biologicals, CO, USA), Alexa Fluor 488 conjugated NLRP3 antibody (1:100, R&D Systems, USA) or ASC antibody (1:100, Cell Signaling Technology) at 4 °C for 60 min, then washed by PBS. Finally, we used DAPI (Beyotime, China) to counterstain the slides and used a confocal microscope (Leica, Wetzlar, Germany) to analyze and photograph.

### Co-immunoprecipitation (Co-IP) and identification of proteins by mass spectrometry (MS)

U2OS cells were collected for cytolysis by lysis buffer (50 mM Tris-HCl pH 7.5, 150 mM NaCl, 5 mM EDTA, and protease inhibitor on ice for 30 min. Following centrifugation, 1 mL lysates were incubated with 1 μg antibodies, USP7 (1:100, Abcam), and then mixed with 10 μL protein A/G agarose for 2 h at 4 °C. The IgG antibody was utilized as a control. The sepharoses were produced by centrifuging at 3000 rpm for 3 min at 4 °C, which were denatured with 15 μL 2 × SDS loading buffer and boiled for 5 min. The combined proteins were analyzed by SDS-PAGE and western blotting. The distinct bank of the SDS-PAGE gel was excised and subjected to in-gel tryptic digestion. After that, we made use of a quadrupole time-of-flight (QTOF) mass spectrometer to perform the digestion of the peptides.

### Terminal deoxynucleotidyl transferase (TdT) dUTP Nick-End Labeling (TUNEL) staining

U2OS cells were downregulated with shUSP7 transfection for 24 h at 37 °C. We fixed U2OS cells with in 4% paraformaldehyde. 0.5% Triton X‐100 solution was used for the pretreatment of cells at room temperature for 5 min, and then stained with TUNEL solution at 37 °C for 60 min. Images were obtained by a fluorescence microscope (Leica, Wetzlar, Germany).

### RT-qPCR, MeRIP-qPCR

Total RNA was isolated from OS tissues or cells by TRIzol (Invitrogen). We used the SYBR Green PCR kit (Qiagen, USA) to perform RT-qPCR following the manufacturer’s protocol. The amplification program was set to 50 °C for 2 min, 95 °C for 10 min followed by 50 cycles of 95 °C for 15 s, 60 °C for 30 s, 72 °C for 5 min. The forward primers of USP7: 5′-AATCATTGGTGTTCATCA-3′, reverse primers of USP7: 5′-CAAGCATCTCATTCTCTT-3′, forward primers of WTAP: 5′-CTGACAAACGGACCAAGTAATG-3′, reverse primers of WTAP: 5′-AAAGTCATCTTCGGTTGTGTTG-3′, forward primers of Raptor: 5′-ACTGAGACGCAACGCCAAAG-3′, reverse primers of Raptor: 5′-GACTTGACGATGATTCCCGC-3′, forward primers of NLRP3: 5′-ATGTGGGGGAGAATGCCTTG-3′, reverse primers of NLRP3: 5′-TTGTCTCCGAGAGTGTTGCC-3′, forward primers of β-actin: 5′-GCATCACACCTTCTACAACGAGC-3′, reverse primers of β-actin: 5′-TCTTCTCCCTGTTGGCTTTGG-3′. The mRNA levels were quantified using the ABI 7500 system (Applied Biosystems, USA). The relative expression of mRNA were calculated using the 2^−ΔΔCt^ method. For MeRIP-qPCR, RNA was incubated with an m^6^A antibody for immunoprecipitation according to the protocol of the Magna MeRIP™ m6A Kit (Millipore, MA, USA). Briefly, the magnetic beads was incubated with m^6^A antibody at 4 °C for 2 h, followed by incubation with 200 μg extracted RNA at 4 °Cfor 3 h. RNA was eluted with 100 μl Elution Buffer (75 nM NaCl, 50 nM Tris-HCl, pH 7.5, 6.25 nM EDTA, 1% (w/v) SDS, 20 mg/ml Proteinase K) for 30 min at 4 °C. Enrichment of m6A was analyzed using qPCR.

### mRNA stability

Stability of RNA in OS cells transfected with shRaptor or Raptor vector was achieved by incubating cells with actinomycin D (Sigma) at 0.5 mg/mL. Cells were then collected at 0–4 h and RNA was isolated for qPCR. USP7 mRNA was detected and normalized using β-actin.

### Luciferase reporter assay

To evaluate the effect of m^6^A on CDS of USP7 expression, the wild type or mutant of CDS of USP7 was inserted behind the F-luc coding region. Both the pmirGLO-USP7-CDS-WT and pmirGLO-USP7-CDS-MUT were transfected into vector or Raptor treated cells for 24 h. The firefly luciferase (F-luc) and renilla luciferase (R-luc) were assayed by Dual-Glo Luciferase Assay system (Promega). The activity of Renilla Luciferase (R-luc) was used to normalize the activity of Firefly Luciferase (F-luc) to evaluate reporter transcription.

### CRISPR/Cas9 knockout

Small guide RNA (sgRNA) sequences targeting the exon 2 of TSC2 and exon 1 of USP7 were cloned into the PX458 vector (Addgene) with the guide RNAs listed below. TSC2: CACCGAACAATCGCATCCGGATGAT, USP7: GTACATGATGCCAACCGAGG. 2 × 10^6^ of U2OS cells were resuspended in transfection medium, and then 2 mg of TSC2 or USP7 sgRNA plasmid was added. Single cell culture and expansion were performed using flow cytometry to select monoclones. The cell colonies were subcultured and a part of each clone was collected for PCR amplification and sequencing to screen clones with TSC2 or USP7 knockout.

### In vivo subcutaneous tumor and metastasis experiments

BALB/c nude mice aged 4-6 weeks were used to perform nude mouse xenograft model. Twenty mice in total were divided into four groups at random: USP7 knockout mice and wild type (WT) mice or shRaptor and shCtrl mice. The flank of the nude mice were subcutaneously injected with 1 × 10^6^ of USP7 KO and control U2OS or shRaptor and shCtrl cells. Measured the tumors’ length and width every 7 days to determine the tumor volume and calculated length × width × width/2 as the tumor volume. After subcutaneous injection of USP7 KO or shRaptor treated cells for 35 days, the tumors were weighed and the figure was taken. The Institutional Animal Care and Use Committee of Zhejiang University gave its approval to the animal protocols.

### Immunohistochemistry (IHC)

We fixed the OS tissues or normal tissues by 4% paraformaldehyde, next dehydrated using graded ethanol solutions, embedded in paraffin, cut into obtain 5 μm sections. Stained the sections with anti-CYP19a1 (1:200, Proteintech, USA) or anti-Ki67 (1:100, Abcam) antibody. Immunostaining was visualized with the DAB staining system (Beyotime). These slices were photographed using a microscope system (Leica, Wetzlar, Germany) and analyzed.

### Statistical analysis

Representative data from at least three biological experiments were presented as mean ± SD. SPSS 17.0 software (SPSS Inc., Chicago, IL, USA) and GraphPad Prism (v9.0, GraphPad Software Inc., San Diego, CA, USA) were applied for analyzing statistics. The data were compared using Student’s *t*-tests or ANOVAs with Tukey’s test. A difference of *P* < 0.05 was regarded as statistically significant.

### Supplementary information

Supplemental Table 1

Supplementary Figure 1

uncropped blots

## Data Availability

Data supporting present findings are available from the corresponding author upon reasonable request.
