# Supplementary material for: mTORC1 accelerates osteosarcoma progression via m6A-dependent stabilization of USP7 mRNA
Source: Cell Death Discov. 2024 Mar 11;10:127. doi: 10.1038/s41420-024-01893-9 (PMC10928159; doi:10.1038/s41420-024-01893-9)
Supplement: Supplementary file 2 — Supplementary Figure 1 [file 41420_2024_1893_MOESM2_ESM.docx]

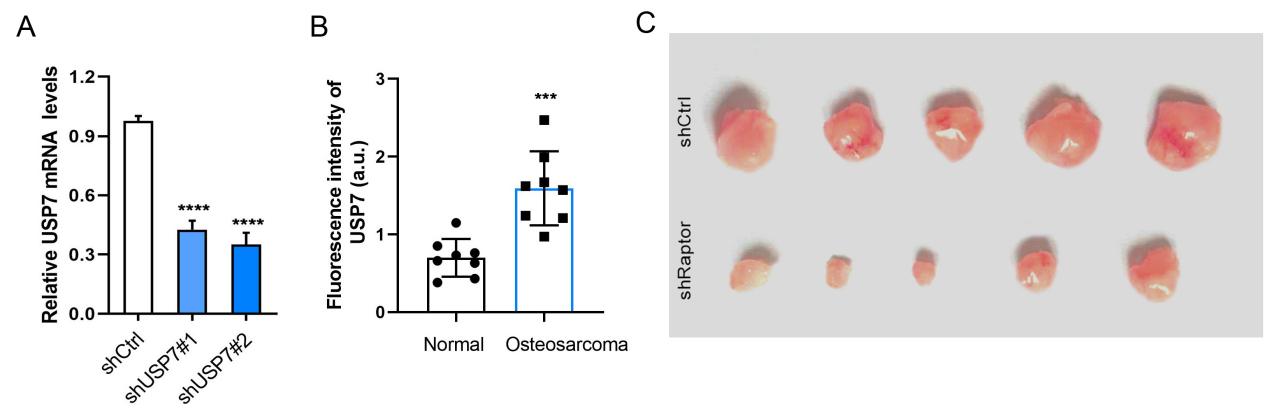


**Supplementary Figure 1. The fluorescence intensity of USP7 was enhanced in OS*.*** (A) The relative mRNA expression of USP7 was determined following shCtrl, shUSP7#1, and shUSP7#2 transfection in U2OS cells. (B) The fluorescence intensity of USP7 was determined in OS patient. (C) The flank of the nude mice were subcutaneously injected with 1 × 10^6^ of shCtrl or shRaptor treated U2OS cells. *** *P* < 0.001, **** *P* < 0.0001.
