# Supplementary material for: mTORC1 accelerates osteosarcoma progression via m6A-dependent stabilization of USP7 mRNA
Source: Cell Death Discov. 2024 Mar 11;10:127. doi: 10.1038/s41420-024-01893-9 (PMC10928159; doi:10.1038/s41420-024-01893-9)

figure 1A P-S6 MG63 figure1B P-S6 U2OS


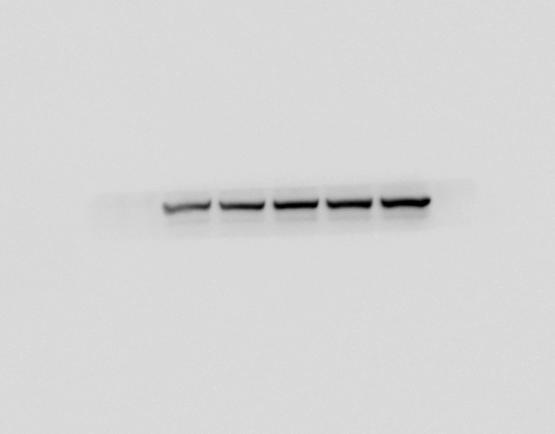

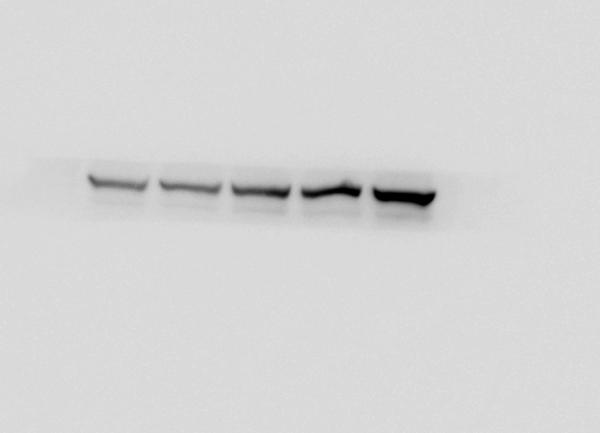


figure 1A p-s6k MG63 figure 1B p-s6k U2OS


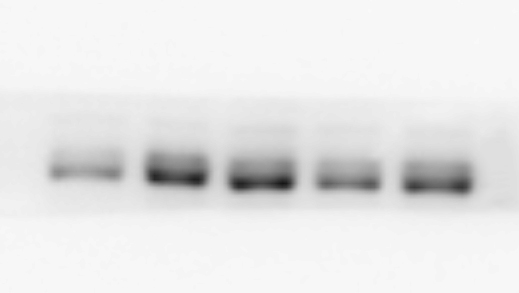

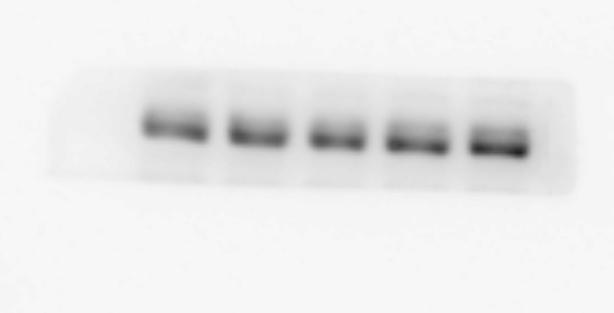


figure 1A S6 MG63 figure 1B S6 U2OS


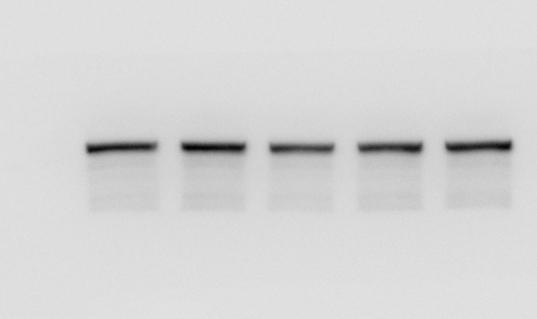

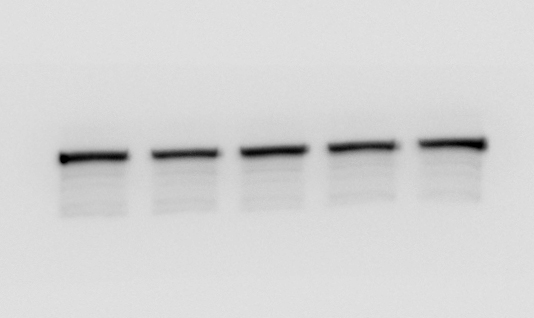


figure 1A s6k MG63 figure 1B s6k U2OS


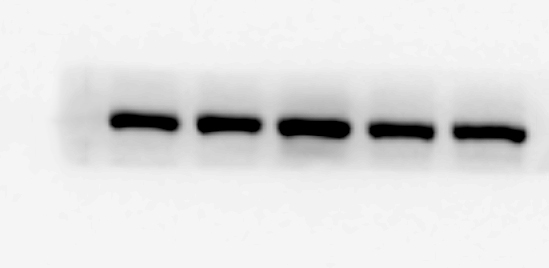

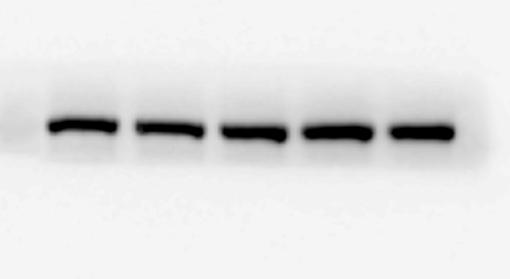


figure 1A β-actin MG63 figure 1B β-actin U2OS


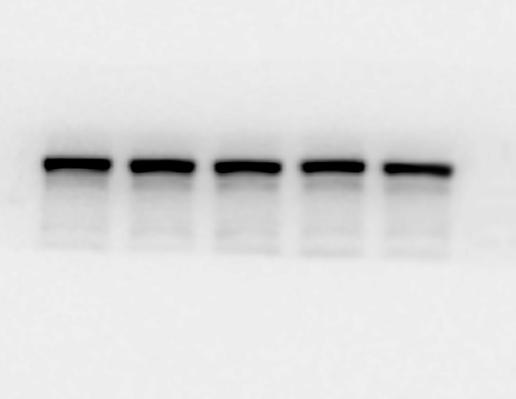

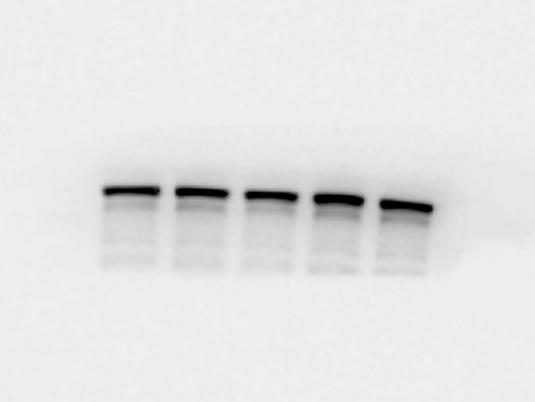


figure 1C MTTLE3 figure 1D MTTLE3


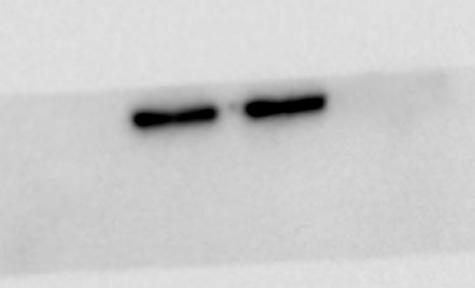

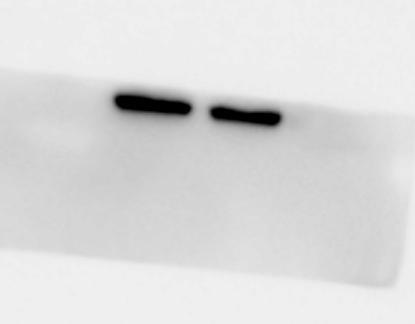


figure 1C MTTLE14 figure 1D MTTLE14


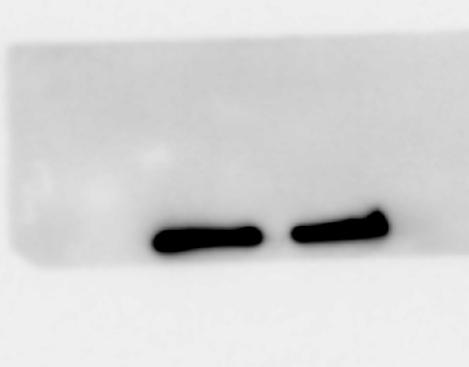

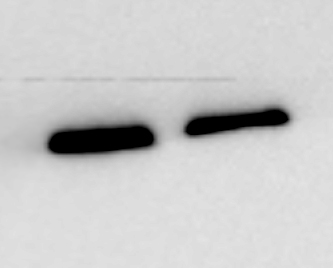


figure 1C WTAP figure 1D WTAP


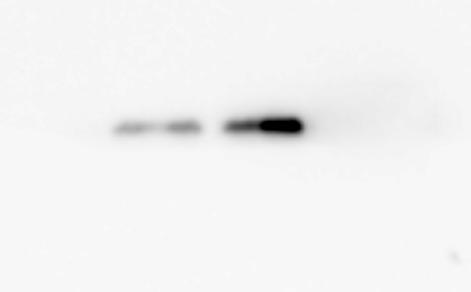

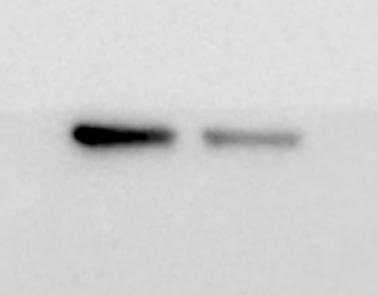


figure 1C β-actin figure 1D β-actin


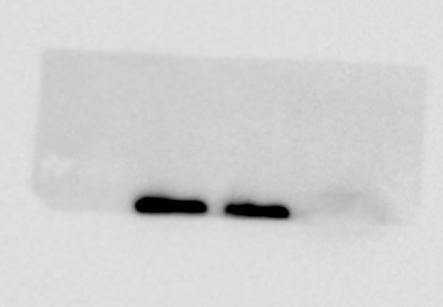

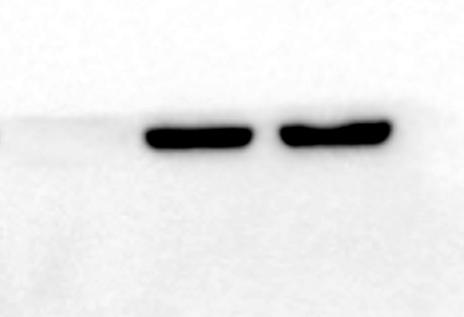


figure 2A WTAP figure 2A β-actin


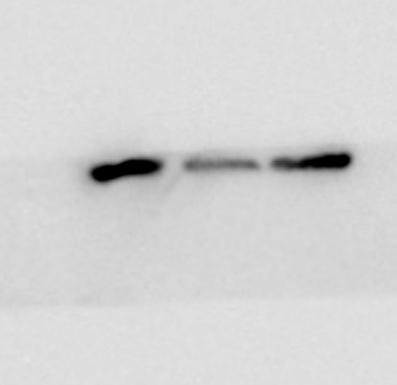

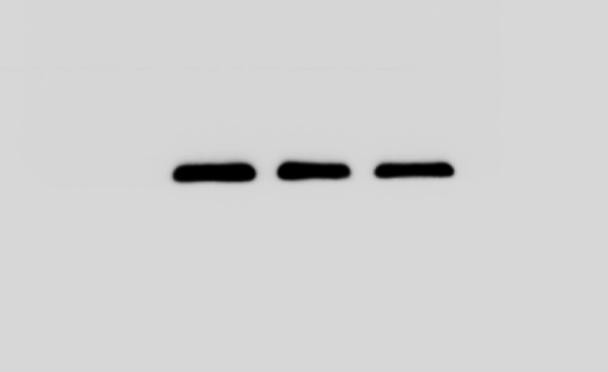


figure 2B Raptor figure 2B p-S6


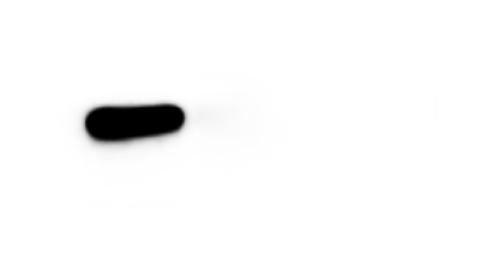

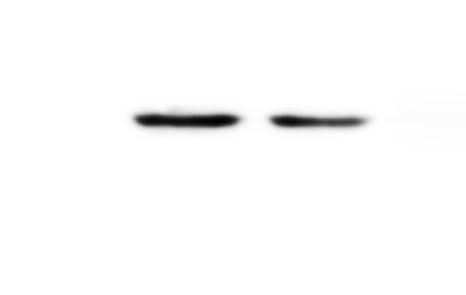


figure 2B S6 figure 2B p-S6K


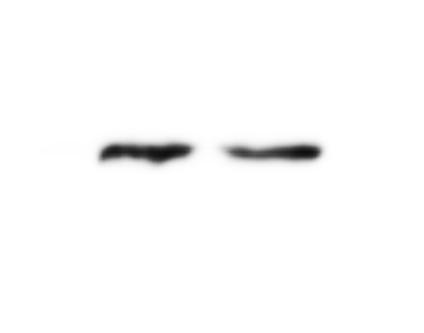

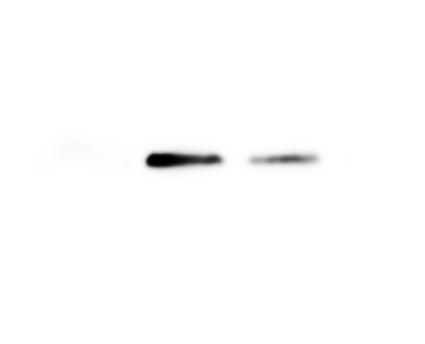


figure 2B S6K figure 2B β-actin


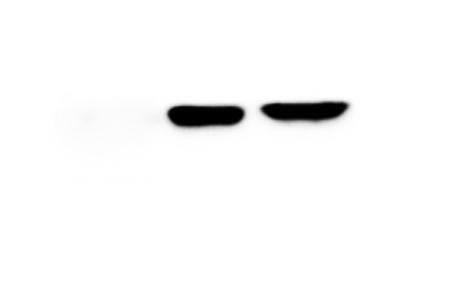

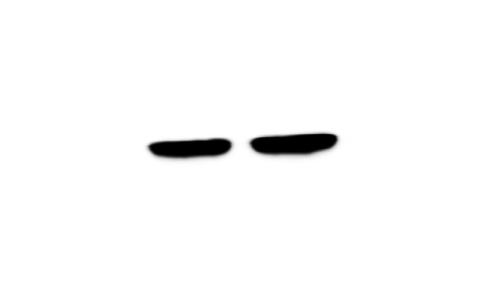


figure 2H TSC2 figure 2H p-S6


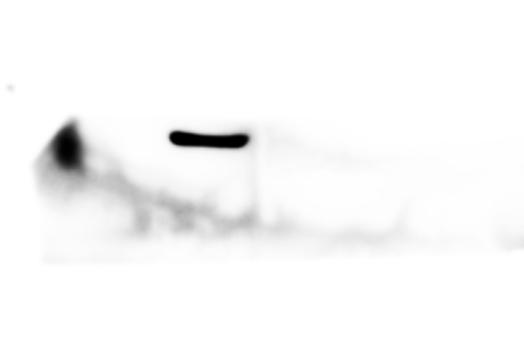

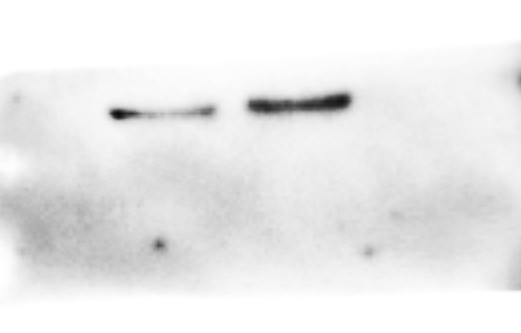


figure 2H S6 figure 2H p-S6K


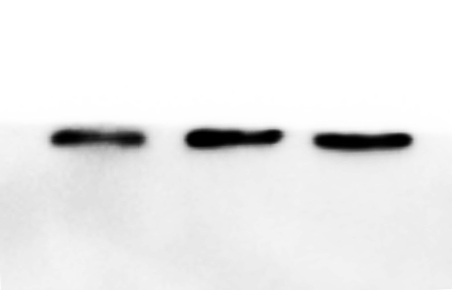

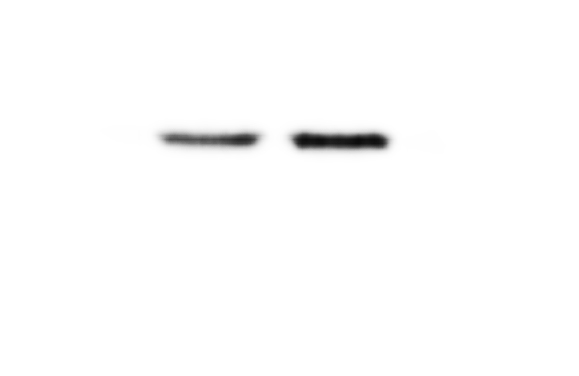


figure 2H S6K figure 2H β-actin


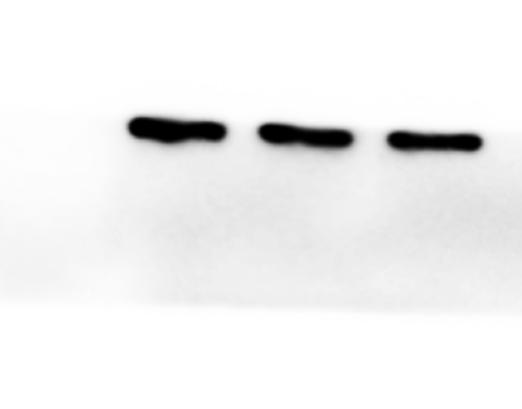

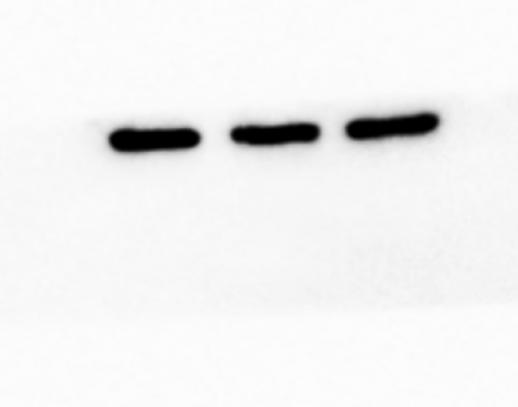


figure 3C p-S6 figure 3C S6


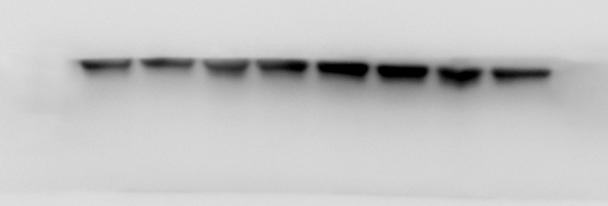

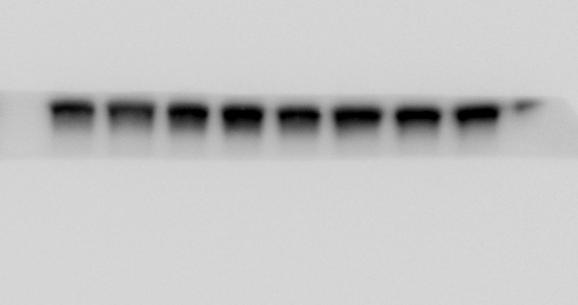


figure 3C β-actin figure 4J Cyp11a1


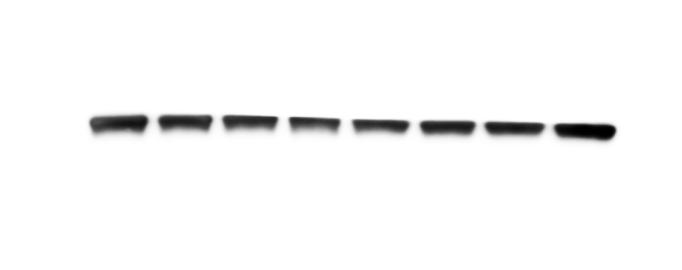

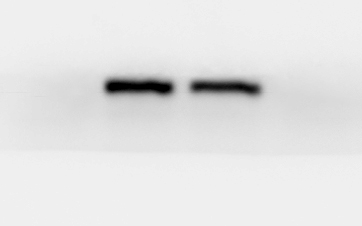


figure 4J Cyp19a1 figure 4J β-actin


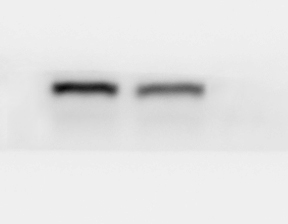

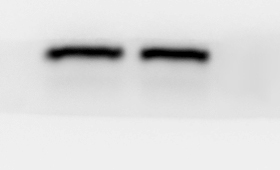


figure 5A IP figure 5B NLRP3 figure 5B USP7


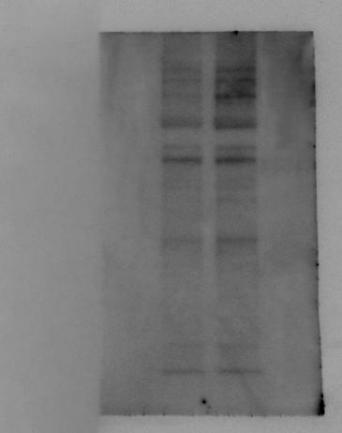

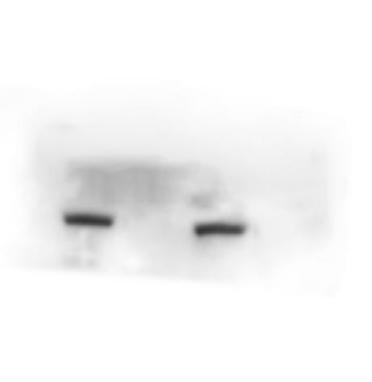

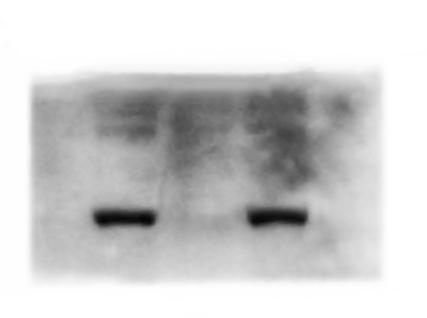


figure 5C caspase1 figure 5C NLRP3


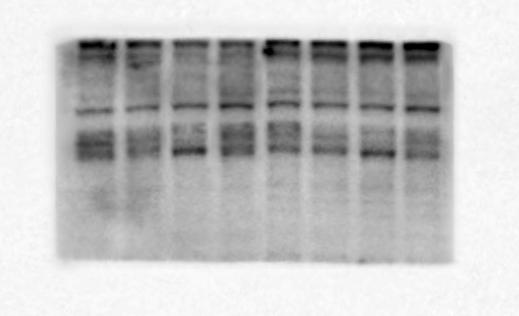

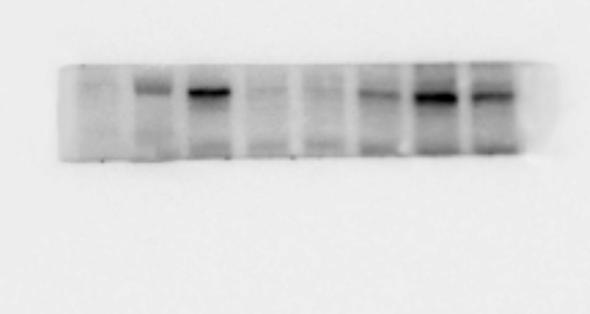


figure 5C β-actin figure 5D β-actin


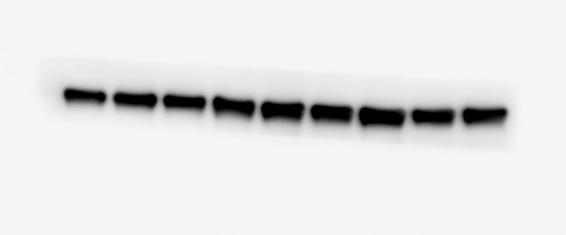

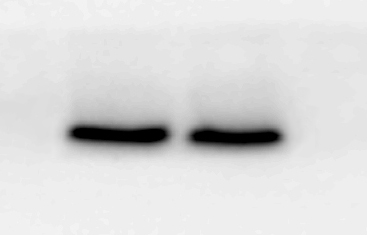


figure 5D NLRP3 figure 5D USP7


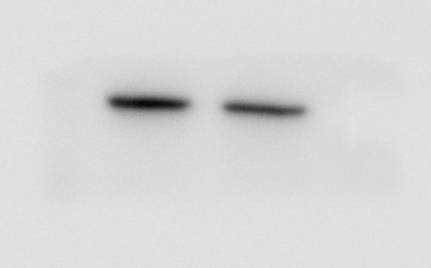

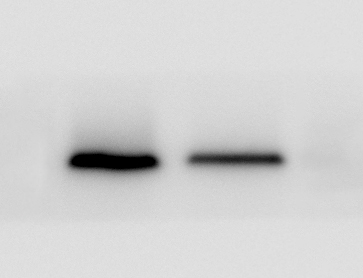


figure 5E K48 figure 5E K63


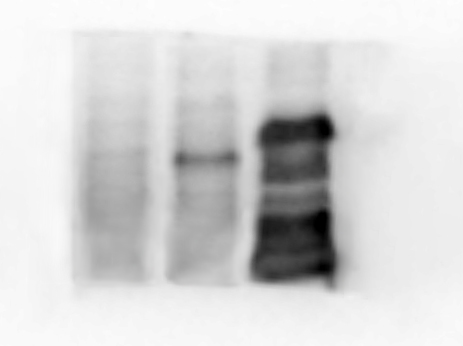

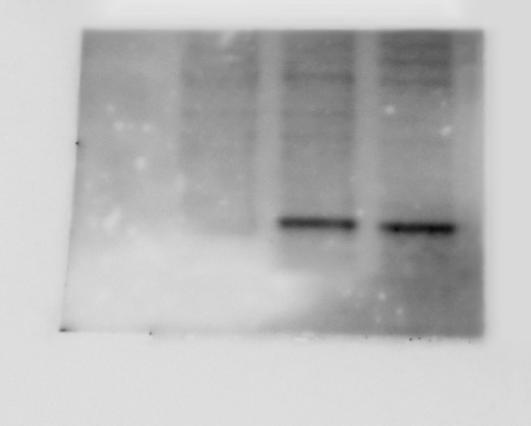


figure 5E NLRP3 INPUT figure 5E NLRP3 IP


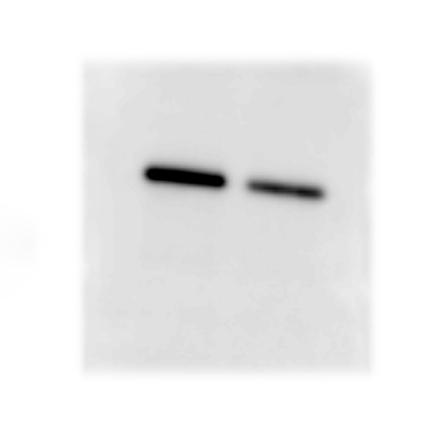

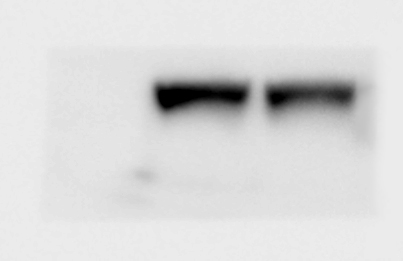


figure 5E USP7 figure 5E β-actin


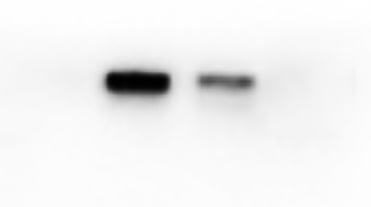

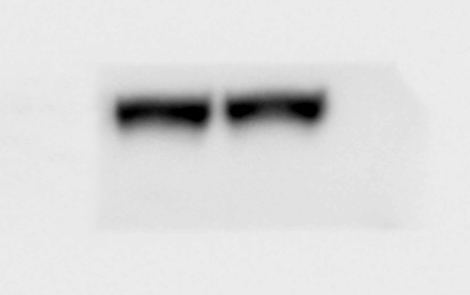

Supplement: Supplementary file 3 — uncropped blots [file 41420_2024_1893_MOESM3_ESM.docx]
